# Impact of Age on Early CAR T‐Cell Therapy Toxicity: A Propensity Score Matching Analysis

**DOI:** 10.1002/cam4.71344

**Published:** 2025-10-29

**Authors:** Jia Yi Tan, Yong‐Hao Yeo, Qi Xuan Ang, George Chen, Kok Hoe Chan

**Affiliations:** ^1^ Department of Internal Medicine New York Medical College at Saint Michael's Medical Center Newark New Jersey USA; ^2^ Department of Internal Medicine/Pediatrics Beaumont Health Royal Oak Michigan USA; ^3^ Department of Internal Medicine Sparrow Health System and Michigan State University East Lansing Michigan USA; ^4^ MD Anderson Cancer Center Houston Texas USA; ^5^ Charleston Area Medical Center Charleston West Virginia USA

**Keywords:** CAR T therapy, chimeric antigen receptor, hematology, oncology, outcomes

## Abstract

**Introduction:**

Despite the growing use of CAR‐T therapy, adults over 65 still receive this treatment less frequently than younger patients.

**Methods:**

Using the Nationwide Readmissions Database (2018–2020), we analyzed 2928 CAR‐T recipients, dividing them into young (18–40), middle‐aged (41–65), and older adults (≥ 66). Propensity score matching (caliper of 0.2, 1:1:1 ratio) was performed. We adjusted for the following confounding variables: gender, comorbidities, and social factors including smoking, alcohol use, and illicit drug use.

**Results:**

Older adults had the highest rates of acute kidney injury (11.7% vs. 13.0% vs. 18.1%, *p* = 0.02) and cardiac complications (2.0% vs. 3.6% vs. 5.4%, *p* = 0.03). These three different age groups had comparable rates of leukopenia (45.0% vs. 42.7% vs. 39.1%, *p* = 0.10), infection (41.0% vs. 43.6% vs. 42.1%, *p* = 0.74), neurotoxicity (6.2% vs. 6.5% vs. 7.7%, *p* = 0.52), and pulmonary embolism (1.0% vs. 2.9% vs. 2.3%; *p* = 0.2). Despite the highest rates of non‐home discharge among the older patients (14.0% vs. 7.5% vs. 8.8%), there were no significant differences in early mortality (5.2% vs. 6.2% vs. 6.7%, *p* = 0.34), 30‐day readmission (23.1% vs. 23.8% vs. 24.4%, *p* = 0.48), prolonged index hospitalization (96.1% vs. 94.8% vs. 93.6%, *p* = 0.14), and total length of stay (21.2 days vs. 18.2 days vs. 21.3 days, *p* = 0.58).

**Conclusion:**

CAR‐T therapy is safe among older adults with close monitoring for cardiac and renal complications.

## 1 Introduction

CAR T‐cell therapy has shown significant efficacy in treating hematologic malignancies, particularly B‐cell malignancies such as acute lymphoblastic leukemia (ALL), non‐Hodgkin lymphoma (NHL), and multiple myeloma (MM). Despite the increasing availability of chimeric antigen receptor T‐cell (CAR‐T) therapy in recent years, adults aged above 65 years still receive this treatment at lower rates than their younger counterparts [1]. Older adults often present with unique challenges, including a higher prevalence of comorbidities, altered immune function, and increased susceptibility to therapy‐related toxicities. Although multiple studies have shown that CAR‐T therapy is safe in older adults [2, 3], large‐scale data on the impact of age on patients receiving CAR‐T therapy are scarce. Our study aimed to investigate the impact of age on hospital outcomes among patients who received CAR‐T therapy to optimize treatment strategies and improve outcomes across all age groups.

## 2 Materials and Methods

National Readmission Database (NRD) is one of the nation's largest publicly available all‐payer inpatient care databases. It is an annual database that includes approximately 17 million discharges per year. Using the International Classification of Diseases, Tenth Revision, Clinical Modification (ICD‐10‐CM), we searched for all adults 18 years of age or above who received CAR‐T therapy between January and September from 2018 to 2020. The study cohort was further categorized into three age groups, namely young adults (18–40 years old), middle‐aged adults (41–65 years old), and older adults (66 years old and above). The in‐hospital outcomes including non‐home discharge (discharge to skilled nursing facility, rehabilitation center, and assisted living facility), prolonged index hospitalization (> 7 days), 30‐day readmission, and early mortality and complications, including acute kidney injury, cardiac complications including acute heart failure, acute myocardial infarction, cardiac arrest, cardiogenic shock, and hypertensive crisis, leukopenia, neurotoxicity, pulmonary embolism, and infection, were compared among these three groups. The propensity score matching was performed with a caliper of 0.2 with a nearest‐neighbor 1:1:1 ratio using R studio. Patients in the middle‐aged group with the closest propensity scores within the specified caliper were matched to patients in the younger age group. The same process was repeated in matching patients in the older age group to the younger age group. This ensured all three groups had similar baseline characteristics listed in Table 1.

**TABLE 1:** Baseline characteristics of patients who received CAR T therapy among 3 different age groups.

	Before propensity score matching				After propensity score matching			
	Age group 1, *n*	Age group 2, *n*	Age group 3, *n*	*p*	Age group 1, *n*	Age group 2, *n*	Age group 3, *n*	*p*
No. of patients	308	1473	1147	—	307	307	299	—
Age, mean (SD), y	29.7 (±6.9)	56.8 (±6.3)	71.8 (±4.4)	—	29.7 (±6.9)	55.6 (±6.7)	71.4 (±4.2)	—
Female	126	549	421	—	126	203	131	—
Alcohol abuse	0	< 10	< 10	0.29	0	0	0	0.00
Anemia	0	16	15	0.14	0	0	0	0.00
Chronic kidney disease	12	118	131	0.00	11	20	18	0.23
Chronic liver disease	16	59	43	0.52	15	24	18	0.52
Chronic pulmonary disease	20	133	108	0.27	20	30	23	0.27
Coagulation disorder	71	303	252	0.52	70	75	35	0.52
Congestive heart failure	< 10	31	51	0.00	< 10	< 10	< 10	0.00
Coronary artery disease	0	68	139	0.00	0	0	< 10	0.00
Diabetes mellitus	16	199	170	0.00	16	23	26	0.00
Hyperlipidemia	11	298	414	0.00	11	18	17	0.00
Hypertension	47	549	588	0.00	47	67	45	0.00
Obesity	24	98	48	0.01	24	32	19	0.01
Obstructive sleep apnea	< 10	96	87	0.01	< 10	14	10	0.01
Peripheral arterial disease	11	36	54	0.01	10	10	< 10	0.01
Prior myocardial infarction	< 10	29	35	0.01	< 10	< 10	< 10	0.01
Prior percutaneous coronary intervention	0	22	49	0.00	0	0	< 10	0.00
Prior coronary artery bypass graft	0	< 10	20	0.00	0	0	0	0.00
Prior stroke/Transient ischemic attack	< 10	23	38	0.00	< 10	< 10	< 10	0.00
Presence of implantable cardioverter defibrillator	< 10	< 10	< 10	0.38	0	< 10	< 10	0.38
Pulmonary hypertension	< 10	34	30	0.87	< 10	15	14	0.87
Smoking	53	406	38	0.00	53	47	48	0.00
Substance use disorder	11	33	< 10	0.00	10	12	< 10	0.00
Valvular heart disease	< 10	30	54	0.00	< 10	< 10	< 10	0.00

*Note:* n, number of admissions.

## 3 Results

Our study included 2928 patients admitted for CAR T‐cell therapy during the study period, which included 308 young adults (10.5%, average age 29.7 ± 6.9 years), 1473 middle‐aged adults (50.3%, average age 56.8 ± 6.3 years), and 1147 older adults (39.2%, average age 71.8 ± 4.4 years). Among young adults, 56.5% were diagnosed with non‐Hodgkin lymphoma (NHL), 29.9% with acute lymphocytic leukemia (ALL), 2.6% with multiple myeloma (MM), and 11.0% with unspecified malignancy. Among middle‐aged adults, 72.8% were diagnosed with NHL, 15.3% with MM, 1.6% with ALL, and 10.3% with unspecified malignancy. Among older adults, 79.1% were diagnosed with NHL, 11.9% with MM, 0.5% with ALL, and 8.5% with unspecified malignancy.

**FIGURE 1:**
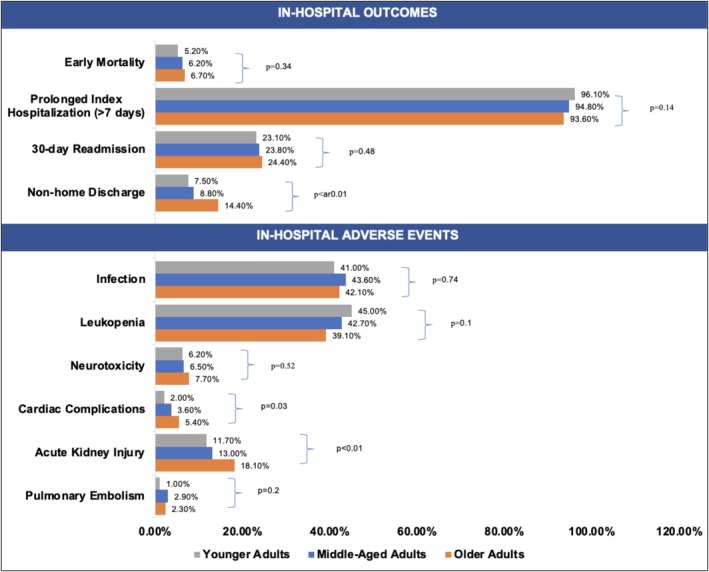
Rates of in‐hospital outcomes and adverse events among patients receiving CAR T‐cell therapy across three different age groups.

After propensity score matching as shown in Table 1, there are 307 young adults, 307 middle‐aged adults, and 299 older adults. In young adults, 56.4% had NHL, 30.0% had ALL, 2.6% had MM, and 11.1% had unspecified malignancy. In middle‐aged adults, 65.8% had NHL, 17.3% had MM, 2.3% had ALL, and 14.7% had unspecified malignancy. In older adults, 79.3% were diagnosed with NHL, 12.7% with MM, 0.3% with ALL, and 7.7% with unspecified malignancy. There were no significant differences in early mortality (young adults, 5.2% vs. middle‐aged adults, 6.2% vs. older adults, 6.7%, *p* = 0.34), 30‐day readmission (23.1% vs. 23.8% vs. 24.4%, *p* = 0.48), and total length of stay (21.2 [±16.6 days] vs. 18.2 [±11.4 days] vs. 21.3 [±16.0 days], *p* = 0.58). Older patients had higher rates of non‐home discharge at 14.0% compared to young and middle‐aged adults at 7.5% and 8.8%, respectively. These three different age groups had comparable rates of leukopenia (45.0% vs. 42.7% vs. 39.1%, *p* = 0.10), infection (41.0% vs. 43.6% vs. 42.1%, *p* = 0.74), neurotoxicity (6.2% vs. 6.5% vs. 7.7%, *p* = 0.52), and pulmonary embolism (1.0% vs. 2.9% vs. 2.3%; *p* = 0.2). Older adults had higher rates of acute kidney injury (11.7% vs. 13.0% vs. 18.1%, *p* = 0.02) and cardiac complications (2.0% vs. 3.6% vs. 5.4%, *p* = 0.03) (Figure 1).

## 4 Discussion

This retrospective cohort study provides important insights into the impact of age on hospital outcomes of CAR‐T therapy. There are no significant differences in early mortality among different age groups, which aligns with one previous study by Ram et al. [2] This is further strengthened by another study by Tun et al. which found that the non‐relapse mortality rates after receiving CAR T‐cell therapy among older adults with relapsed or refractory large B‐cell lymphoma were not associated with age groups [4]. In contrast, Lemoine et al. reported an increased incidence of non‐relapse mortality within 28 days among elderly patients with a median age of 73 [5]. Despite this, our study suggests that age is not a contraindication to CAR‐T therapy. Our study shows that older adults have a higher risk for cardiac complications following CAR‐T therapy. This is consistent with a study by Steiner et al. which demonstrates that patients aged 60 years and older were significantly associated with an increased risk of major adverse cardiovascular events (MACE) within 30 days of CAR T‐cell therapy [6]. Such a phenomenon can be explained by the increased susceptibility among older adults to cytokine release syndrome, which was associated with increased adverse cardiac events [7, 8]. Additionally, older patients usually had a decrease in cardiac reserve and an increased vulnerability to stressors [7]. Older patients were found to be at higher risk of developing high‐grade cytokine release syndrome, which is a significant risk factor for acute kidney injury [9, 10].

Our study is not without limitations. First, specific patient variables such as the severity of comorbidities and the type of CAR‐T therapy administered are unavailable. Second, our study relies heavily on the accuracy and availability of ICD coding. The common complications associated with CAR T‐cell therapy, including cytokine release syndrome and immune effector cell‐associated neurotoxicity syndrome were not analyzed as their ICD‐10 codes are only available in 2021. Third, NRD is an inpatient database. Therefore, it might underestimate the risk of complications, which may be treated in the outpatient setting. Despite these limitations, our study demonstrates no significant differences in hospital outcomes, including early mortality, early readmission, and length of stay among young, middle‐aged, and older adults following CAR T‐cell therapy. In‐hospital complications such as leukopenia, infection, neurotoxicity, and pulmonary embolism were comparable across all age groups. These findings suggest that CAR T‐cell therapy is safe for older patients. However, increased vigilance for cardiac complications and acute kidney injury is crucial when managing older patients undergoing CAR T‐cell therapy.

## Author Contributions

**Jia Yi Tan:** conceptualization, writing – original draft, project administration, visualization. **Yong‐Hao Yeo:** methodology, investigation, formal analysis, data curation. **Qi Xuan Ang:** software, formal analysis. **George Chen:** writing – review and editing. **Kok Hoe Chan:** writing – review and editing.

## Ethics Statement

This research study was conducted retrospectively from data obtained from HCUP. It does not require institutional review approval as the population data is de‐identified.

## Consent

The population data on HCUP is de‐identified. All authors gave consent for the submission of the manuscript.

## Conflicts of Interest

The authors declare no conflicts of interest.

## Data Availability

The data that support the findings of this study are openly available in Healthcare Cost and Utilization Project at https://www.ahrq.gov/data/hcup/index.html.
